# Technology‐natives and technology‐adopters: Age differences in remote cognitive assessment in Alzheimer disease

**DOI:** 10.1002/alz.71872

**Published:** 2026-09-24

**Authors:** Samhita Katteri, Hannah Wilks, Matthew Welhaf, Andrew J. Aschenbrenner, Colleen C. Frank, Clara Vila‐Castelar, Sarah Stout, Martin J. Sliwinski, Tammie LS Benzinger, Ma. Florencia Clarens, Carlos Cruchaga, Alisha Daniels, Gregory S Day, Emma Devenney, Brian A. Gordon, Edward D. Huey, Takeshi Ikeuchi, Takanobu Ishiguro, Laura Ibanez, Mathias Jucker, Celeste Karch, Johannes Levin, Richard Perrin, Alan Renton, Charlene Supnet‐Bell, Philip S. J. Weston, Chengjie Xiong, Jorge Libre‐Guerra, Eric M. McDade, Randall J. Bateman, John C. Morris, Dominantly Inherited Alzheimer Network, Jason Hassenstab

**Affiliations:** ^1^ Department of Psychological & Brain Sciences Washington University in St. Louis St. Louis Missouri USA; ^2^ Department of Psychology North Carolina A&T State University Greensboro North Carolina USA; ^3^ Department of Neurology University of Kansas Alzheimer's Disease Research Center University of Kansas Medical Center Kansas City Missouri USA; ^4^ Charles F. and Joanne Knight Alzheimer Disease Research Center Department of Neurology Washington University School of Medicine St. Louis Missouri USA; ^5^ Department of Human Development and Family Studies Pennsylvania State University State College Pennsylvania USA; ^6^ Department of Radiology Washington University School of Medicine St. Louis Missouri USA; ^7^ Department of Cognitive Neurology Instituto Neurológico Fleni Buenos Aires Argentina; ^8^ Department of Psychiatry Washington University School of Medicine St. Louis Missouri USA; ^9^ Department of Neurology Mayo Clinic Jacksonville Florida USA; ^10^ Neuroscience Research Australia Sydney Australia; ^11^ Faculty of Medicine and Health University of New South Wales Sydney Australia; ^12^ Department of Psychiatry and Human Behavior Alpert Medical School of Brown University Providence Rhode Island USA; ^13^ Brain Research Institute Niigata University Niigata Japan; ^14^ German Center for Neurodegenerative Diseases (DZNE) Tübingen Germany; ^15^ Hertie‐Institute for Clinical Brain Research University of Tübingen Tübingen Germany; ^16^ Department of Neurology Ludwig‐Maximilians Universität München Munich Germany; ^17^ German Center for Neurodegenerative Diseases (DZNE) Munich Germany; ^18^ Munich Cluster of Systems Neurology (SyNergy) Munich Germany; ^19^ Department of Pathology & Immunology Washington University School of Medicine St. Louis Missouri USA; ^20^ Dementia Research Centre UCL Queen Square Institute of Neurology London UK; ^21^ UK Dementia Research Institute London UK; ^22^ Department of Biostatistics Washington University School of Medicine St. Louis Missouri USA

**Keywords:** Alzheimer disease, digital remote cognitive assessment, early detection, older adults, technology familiarity, young adults

## Abstract

**INTRODUCTION:**

Cognitive changes in the asymptomatic stages of Alzheimer disease (AD) are subtle and difficult to detect using conventional cognitive tests. High‐frequency smartphone‐based testing may improve sensitivity to these changes, but generational differences in technology use could influence performance. We examined generational differences in cognition using remote, high‐frequency smartphone assessments.

**METHODS:**

Older adults (*N* = 444) from the Knight Alzheimer Disease Research Center and young and middle‐aged adults (*N* = 113) from the Dominantly Inherited Alzheimer Network (DIAN) completed an in‐clinic cognitive battery and brief smartphone tests up to four times daily for 7 days. We compared adherence, reliability, practice effects, and correlations with in‐clinic measures.

**RESULTS:**

Older adults showed higher adherence than younger participants (78% vs 59%). Younger adults achieved higher reliability with fewer sessions and exhibited much smaller practice effects. Smartphone to in‐clinic test correlations were strong in both young‐to‐middle‐aged and older adults.

**DISCUSSION:**

Generational differences have important implications for the design and implementation of smartphone‐based cognitive studies.

## Highlights

- High‐frequency smartphone testing enables reliable cognitive assessments across ages in populations at risk for dementia.
- The correlation between smartphone‐based tests and traditional in‐clinic tests was strong in both young‐to‐middle‐aged and older adults.
- Older adults demonstrated strong effects of practice on smartphone‐based tests but these effects were marginal in younger adults.
- Both age and technology familiarity influence usage patterns and performance on digital cognitive measures.

## 1 BACKGROUND

Alzheimer disease (AD) develops gradually, with a long preclinical stage that can span two decades or more before symptoms emerge.^1^, ^2^ During this preclinical stage there may be subtle changes in cognition. Detecting these very mild changes is challenging, but critical for understanding the nature and timing of disease progression. In the era of disease‐modifying therapies, highly sensitive cognitive tests are also needed to track response to treatments in early stages.^3^

Remote, unsupervised high‐frequency smartphone‐based cognitive assessments are sensitive to subtle changes in cognition and designed to provide near real‐time cognitive data in naturalistic settings.^4^, ^5^, ^6^ Most studies using high frequency smartphone‐based cognitive assessments have focused on validating these tests for older adults who are at risk of developing sporadic AD. These studies have shown tasks to have high between‐person reliability,^7^, ^8^ examined how practice effects differ across tasks^9^ and by cognitive status^7^, and demonstrated concurrent validity between smartphone‐based and in‐clinic cognitive tasks.^7^, ^8^ In contrast, few studies have examined these patterns in younger adults, though emerging evidence supports feasibility^10^, ^11^ and validity of remote cognitive testing in young and middle‐aged adults.^10^

AD not only impacts older adults, but also young‐to‐middle‐aged adults. The median age of onset of sporadic AD is approximately 72 years.^12^ In genetic forms of AD like dominantly inherited AD (DIAD) and Down syndrome AD (DSAD), symptom onset begins much earlier, typically in the 40s and 50s.^1^, ^13^, ^14^ Heterogeneity in the age of symptom onset of AD underscores the need for sensitive cognitive assessments that detect early cognitive changes across the adult lifespan. Therefore, more validation studies are needed among young‐to‐middle‐aged adults at risk for AD to understand whether current designs of high‐frequency cognitive assessments are optimally designed for adulthood across the lifespan.

Age‐related differences in processing speed, technology familiarity, and life stage demands are factors that may influence the validity and feasibility of high‐frequency smartphone‐based cognitive assessments. Age‐related changes in speed of processing affect many aspects of cognition^15^, ^16^ and are especially relevant to smartphone‐based cognitive measures where speed is commonly emphasized. The concept of digital nativity^17^, ^18^ describes younger generations as “digital natives” who grew up with smartphones and therefore use this technology more fluently compared to older generations or “digital immigrants” who did not have exposure to such devices during adolescence, a critical time period where life‐long habits are formed.^19^ Accordingly, older age has been found to be associated with significantly lower levels of technology familiarity compared to young and middle‐aged adults.^11^ However, studies have found that older adults have high adherence for repeated smartphone‐based cognitive assessments and were extremely willing and capable participants.^9^, ^11^ Additionally, younger adults have been found to experience higher work and family conflict compared to older adults due to increased demands in both domains, whereas older adults comparatively face lower conflict as demands from these domains tend to decrease with age.^20^ Taken together, these differences raise important questions about how validity and feasibility of smartphone‐based cognitive assessments may vary across the adult lifespan.

**RESEARCH IN CONTEXT**

1. **Systematic review**: The authors reviewed published research on smartphone‐based cognitive assessments. Although younger populations at risk for Alzheimer disease (AD) have not been widely studied in the smartphone‐based cognitive assessment literature, several publications discuss the validation of such assessments among older adults at risk for AD. These citations are appropriately cited.
2. **Interpretation**: High‐frequency remote cognitive assessment designs produce extremely reliable and valid cognitive data in AD populations of all ages. However, generational differences in technology familiarity appear to influence performance on smartphone‐based cognitive assessments, including adherence, reliability, and learning effects. Older adults benefit from more frequent testing but show large practice effects while younger adults, despite lower adherence, may complete fewer sessions without compromising reliability or validity.
3. **Future directions**: Studies administering high‐frequency smartphone‐based cognitive assessments should carefully consider levels of technology familiarity when interpreting age differences in usage patterns, reliability, and performance.

The current study directly addressed these questions by comparing high‐frequency smartphone‐based cognitive assessments usage patterns, reliability, practice effects, and correlations with conventional measures in two independent samples at risk for AD separated by approximately 40 years in age. Building on prior findings of strong adherence among older adults and considering the difference in life stage demands between the two samples, we hypothesized that older adults would exhibit higher adherence to the smartphone‐based assessment protocol compared to young‐to‐middle‐aged adults. Based on known differences in technology familiarity and age‐related differences in processing speed affecting many aspects of cognition, we hypothesized lower measurement reliability, greater practice effects, and weaker correlations between smartphone‐based and in‐clinic cognitive assessments in the older sample relative to the young‐to‐middle‐aged sample.

## 2 METHODS

### 2.1 Knight ADRC participants

Participants in the older adult sample were recruited from studies of aging and dementia and enrolled into the Ambulatory Research in Cognition (ARC) study at the Charles F. and Joanne Knight Alzheimer Disease Research Center (Knight ADRC) at Washington University School of Medicine in St. Louis. Because the ARC study was intended to describe cognitive dynamics from the asymptomatic preclinical stages to the early symptomatic stage of AD, enrollment was limited to participants with a Clinical Dementia Rating® (CDR®^21^) score of 0—normal cognition—and 0.5—very mild dementia at their previous visit. From this point forward, older adult participants from this sample will be referred to as the Knight ADRC sample.

### 2.2 DIAN Participants

Participants in the young‐to‐middle‐aged adult sample were recruited from the Dominantly Inherited Alzheimer Network observational study (DIAN), which is an international, multisite longitudinal study of families in which approximately 50% of family members carry an autosomal‐dominant Alzheimer disease mutation in either the *amyloid precursor protein* (*APP)*, *presenilin 1* (*PSEN1)*, or *presenilin 2* (*PSEN2)* genes. Mutation carriers refer to family members carrying a confirmed pathogenic mutation, and non‐carriers are family members that do not carry a mutation. Similar to the Knight ADRC study, the DIAN ARC smartphone study is focused on detection of early changes in cognition; therefore, enrollment was limited to mutation carriers and non‐carriers with a global CDR score of 0 and 0.5 at their previous visit. Data were collected at 14 sites that completed ARC testing and included participants from the United States of America, Argentina, Colombia, the United Kingdom, Australia, and Japan. For each participant, an estimated years to symptom onset (EYO) was calculated at each visit. EYO functions as a proxy of disease stage. The EYO was calculated as the participant's age at assessment minus the mutation‐specific mean age at onset, derived from symptomatic carriers within the DIAN sample.^13^, ^22^ Negative EYO values indicate years prior to the expected onset, whereas positive values indicate years after expected onset. For example, a participant with an EYO of ‐3 is 3 years away from the average age when all participants with the same family mutation experienced onset of dementia symptoms. From this point forward, this sample will be referred to as the DIAN sample.

The Knight ADRC and DIAN samples both used the National Alzheimer's Coordinating Center's Uniform Data Set (NACC UDS) forms to collect information regarding sex and years of education. Sex is reported as a binary self‐report measure often collected by the clinician at baseline. Guidelines for the NACC UDS are provided in DIAN to ensure that education is quantified in a consistent manner cross‐culturally. Both Knight ADRC and DIAN participants provided informed consent. All procedures were approved by local institutional review boards.

### 2.3 Clinical assessment

In both samples, clinical status was determined by trained clinicians with the CDR. The CDR is a semi‐structured interview conducted separately with both the participant and an informant (typically a family member or close friend) to stage cognitive and functional deficits across six domains, including memory, orientation, judgment and problem solving, community affairs, home and hobbies, and personal care. A CDR score of 0 indicates normal cognition, while scores of 0.5, 1, 2, and 3 indicate very mild, mild, moderate, and severe dementia, respectively.^21^

### 2.4 Cognitive assessments

The Knight Preclinical Alzheimer's Cognitive Composite (PACC) is a global cognitive composite sensitive to change in the preclinical stages of AD.^23^ The Knight‐PACC, calculated using the Knight ADRC sample, averages the standardized scores from the Free and Cued Selective Reminding Test (FCSRT) free recall,^24^ Animal naming total score,^25^ Trail Making Test Part B total completion time,^26^ and the total correct score from the Number Symbol test, a digital version of the Digit Symbol Substitution test created at the Knight ADRC. The DIAN‐PACC, calculated using the DIAN sample, followed a similar procedure as the Knight‐PACC except for using a paper version of the Digit Symbol test instead and the Logical Memory II Delayed recall^27^ score being substituted for the FCSRT free recall. In the DIAN study, tests were adapted where appropriate for language and culture by following a forward‐backward translation procedure and a verification by local clinicians from each DIAN site.

### 2.5 ARC smartphone application

The ARC smartphone application, used in the Knight ADRC and DIAN studies, was built for iOS and Android devices to administer brief cognitive tests across seven consecutive days, up to four times daily, allowing a maximum of 28 sessions. Notifications were sent during participants’ self‐reported wake hours, pseudorandomly spaced to ensure at least two hours between sessions. Participants had two hours to respond. Based on age being associated with lower technology familiarity^11^, the Knight ADRC sample received technological support from study coordinators by phone or email to inquire about technological issues or when adherence fell below 60%. This structured support was not provided to the younger DIAN sample. For full details on ARC development and psychometrics, see Nicosia et al., 2023.

Each session included three cognitive tasks: Symbols, Prices, and Grids, administered in random order. Briefly, Symbols measures processing speed, lasting 20–60 s; its primary outcome is the median reaction time (RT) on correct trials. Grids measures visual working memory lasting 30–40 s; its primary outcome is a Euclidean distance (higher scores = greater error). Prices measures associative memory with learning and recognition phases, lasting ∼60 s; its primary outcome is the percent errors from 10 recognition trials.

In DIAN, Prices was adapted for language and culture using an internationalization procedure.^28^ Native speakers generated 75 common shopping items priced near $10; duplicates were removed, and items were rated for familiarity by a different set of native speakers. The top 40 items were used, with prices converted to local currency. For currencies like Japanese Yen, the fourth digit was set to zero so participants recalled three‐digit prices consistently.

To compare ARC performance with the Knight‐PACC and DIAN‐PACC, an “ARC composite score” was created. For each sample, Z‐scores for each task were calculated using the sample mean and standard deviation separately, then averaged. To examine practice effects, the ARC composite calculation involved using the grand mean and grand standard deviation across both the ADRC and DIAN samples. For ease of interpretation, scores were multiplied by ‐1 such that higher scores on the composites indicate better cognitive performance.

### 2.6 Statistical analyses

R (v4.4.1) was used to complete statistical analyses. Descriptive statistics were conducted to characterize both samples and ARC and conventional cognitive task performance. Welch's t‐tests and chi‐square tests, when examining categorical variables, were conducted to determine group differences between mutation carrier and non‐carriers in the DIAN sample (Table S1).

We ran a linear regression model to test for differences in adherence by sample, controlling for education, and sex. We ran additional linear regression models to test for differences in adherence within each sample.

An exploratory analysis was conducted to better understand adherence to ARC testing. The ARC session times were categorized as occurring in the morning (5:00 AM ‐ 11:59 AM), afternoon (12:00 PM–4:59 PM), evening (5:00 PM–8:59 PM), or night (9:00 PM–4:59 AM).^29^ A Bayesian multinomial mixed‐effects model was used to examine whether participants in each sample were more likely to complete ARC sessions at certain times of the day. The model adjusted for covariates (education and sex) and included a random intercept term for each participant. Using population‐level predictions, we calculated predicted probabilities, between‐sample differences, 95% credible intervals, and posterior probabilities from the fitted model.

Between‐person reliability provides an estimate of the reliability of the differences between participants when averaged across a given number of testing sessions. Using the lme4 package in R^30^, linear mixed‐effects models were fit using restricted maximum likelihood for each ARC task to estimate intraclass correlation coefficients (ICCs). The model included the ARC task‐specific primary metric as the outcome variable and a random intercept term for each participant. These ICCs assessed between‐person reliability across ARC sessions for each ARC task.^31^, ^32^ The higher the ICC, the more reliable the measure is in distinguishing between participants’ task performance. A “good” reliability is characterized by ICCs between 0.75 and 0.90.^33^ Based on the upper end of this range, we decided that ICCs of 0.90 and higher define excellent reliability.

Practice effects were modeled with the ARC composite. To prevent extreme outliers from skewing the results, participants with an ARC composite of less than ‐3 in any ARC session were removed. Four participants from the Knight ADRC sample were removed in the practice effects analysis, leaving 440 Knight ADRC and 113 DIAN participants. Using the lme4 package^30^ and lmerTest package^34^ to derive *p*‐values, linear mixed effects models with random intercepts and slopes estimated the slopes of the ARC composite across all sessions in each sample, with terms for education, sex, the total number of sessions completed across the week of testing, and the absolute value in years of the date difference between the ARC testing and clinical evaluation dates included as covariates. To test for nonlinear effects, quadratic and cubic terms were considered in models, but model fit indicated that linear models were sufficient. We interpreted the interaction between sample (Knight ADRC and DIAN) and sessions as a marker of practice effects. Using estimated marginal means extracted from the linear mixed effect model, effect sizes were calculated as the unstandardized mean difference in the ARC composite between the Knight ADRC and DIAN samples at the first and last ARC session.

Partial correlations, controlling for education, sex, and the absolute value of the date difference in years between the ARC and conventional cognitive testing were used to describe associations between cognitive measures (ARC and conventional tests). Fishers r‐to‐z tests compared the Knight ADRC and DIAN samples to determine if there were differences in the strength of correlations between ARC tasks with conventional cognitive[Table alz71872-tbl-0001] measures.

## 3 RESULTS

### 3.1 Sample characteristics

#### 3.1.1 Knight ADRC

Four‐hundred forty‐four participants were enrolled from the Knight ADRC in the ARC smartphone study. Table 1 displays the Knight ADRC sample characteristics. All participants were cognitively normal (CDR 0). Participants were on average 75.4 ± 6.1 years old, more likely to be female (56.9%), be highly educated (16.5 ± 2.4), and most self‐reported their race as White (83.6%). See Figure S1 for Knight ADRC demographic, ARC, and in‐clinic Pearson correlations.

**TABLE 1 alz71872-tbl-0001:** Knight ADRC sample demographics data.

Parameter	CDR 0
	*N* = 444^a^
Age	75.39 (6.07)
Sex (% female)^b^	253 (56.9%)
Race^b^	
Black	71 (15.9%)
White	371 (83.6%)
Other	2 (0.5%)
Education	16.47 (2.42)
Grids	*N* = 443
	0.74 (0.27)
Prices	0.29 (0.09)
Symbols	*N* = 443
	3.3 (1.13)
Standardized ARC Composite	0.07 (0.70)
Adherence	78.42% (22.09%)
SRT Free Recall	*N* = 381
	31.93 (5.93)
Category Fluency Animals	*N* = 442
	21.02 (5.44)
Number Symbol	*N* = 381
	39.46 (8.00)
Trail Making Test Part B^c^	*N* = 135
	76.93 (29.77)
Standardized In‐clinic Composite	*N* = 366
	0.10 (0.67)
Time interval between ARC and in‐clinic testing^d^	0.09 (0.24)
	min = 0
	max = 1.23
Time interval between ARC testing and clinical evaluation^d^	0.19 (0.25)
	min = 0
	max = 1.23
Time interval between in‐clinic testing and clinical evaluation^d^	0.13 (0.19)
	min = 0
	max = 1.15
Geriatric Depression Scale	*N* = 154
	1.03 (1.48)
Neuropsychiatric Inventory ‐ Number of Symptoms Endorsed	*N* = 444
	0.50
Neuropsychiatric Inventory ‐ Severity ^e^	*N* = 132
	2.06

Abbreviations: ADRC, Alzheimer Disease Research Center; ARC, Ambulatory Research in Cognition; CDR, Clinical Dementia Rating scale; SRT, Selective Reminding Test.

^a^ Mean (SD); n (%).

^b^ Sex and race were self‐reported.

^c^ Sample sizes were substantially lower due to restrictions on in‐person testing during the COVID‐19 pandemic.

^d^ The time interval is reported in years. It was calculated as the number of days between the two categories divided by 365.

^e^ The sample size for the Neuropsychiatric Inventory – Severity is lower than the Neuropsychiatric Inventory – Number of Symptoms Endorsed since we are only reporting the severity for those who reported any symptoms.

Participants across the Knight ADRC sample received a clinical evaluation within 0.19 ± 0.25 years (range: 0‐1.23 years) of ARC testing. They completed a battery of conventional cognitive tests within 0.09 ± 0.24 years (range: 0‐1.23 years) of ARC testing and within 0.13 ± 0.19 years (range: 0‐1.15 years) of the clinical evaluation.

#### 3.1.2 DIAN

One‐hundred and thirteen participants were enrolled from DIAN in the ARC smartphone study, including 56 mutation carriers (MCs) and 57 non‐carriers (NCs). Table 2 displays the DIAN sample characteristics. All participants were cognitively normal (CDR 0). Participants were on average, 35.8 ± 10.3 years old with an average EYO of ‐15.0, indicating the average participant was approximately 15 years before symptom onset. Participants were more likely to be female (60.2%), have 14.6 ± 2.9 years of education, and most self‐reported their race as White (77%). See Figure S2 for DIAN demographic, ARC, and in‐clinic Pearson correlations.

**TABLE 2:** DIAN sample demographic data.

Parameter	CDR 0
	*N* = 113^a^
Age	35.76 (10.34)
Sex (% female)^b^	68 (60.2%)
Race^b^	
Black	17 (15%)
White	87 (77%)
Other	9 (8%)
Education	14.59 (2.90)
Mutation carrier (%)	56 (49.6%)
Estimated year to onset (EYO)	−15.04 (11.09)
Grids	0.51 (0.25)
Prices	0.25 (0.14)
Symbols	2.27 (0.72)
Standardized ARC Composite	0.17 (0.60)
Adherence	59.3% (29.1%)
Logical Memory II Delayed recall	*N* = 98
	13.38 (4.41)
Category Fluency Animals	*N* = 107
	21.55 (6.13)
Digit Symbol	*N* = 107
	59.85 (17.35)
Trail Making Test Part B	*N* = 105
	63.50 (36.70)
Standardized In‐Clinic Composite	*N* = 107
	0.12 (0.68)
Time interval between ARC and in‐clinic testing^c^	0.18 (0.43)
	min = 0
	max = 2.92
Time interval between ARC testing and clinical evaluation^c^	0.18 (0.43)
	min = 0
	max = 2.92
Time interval between in‐clinic testing and clinical evaluation^c^	0 (0)
	min = 0
	max = 0
Geriatric Depression Scale	*N* = 98
	2.29 (2.21)
Neuropsychiatric Inventory ‐ Number of Symptoms Endorsed	*N* = 113
	0.48 (1.17)
Neuropsychiatric Inventory ‐ Severity ^d^	*N* = 30
	2.57 (3.56)

Abbreviations: ARC, Ambulatory Research in Cognition; CDR, Clinical Dementia Rating scale; DIAN, Dominantly Inherited Alzheimer Network observational study.

^a^ Mean (SD); n (%).

^b^ Sex and race were self‐reported.

^c^ The time interval is reported in years. It was calculated as the number of days between the two categories divided by 365.

^d^ The sample size for the Neuropsychiatric Inventory – Severity is lower than the Neuropsychiatric Inventory – Number of Symptoms Endorsed since we are only reporting the severity for those who reported any symptoms.

In DIAN, the clinical evaluation and conventional cognitive testing was completed within 0.18 ± 0.43 years (range: 0‐2.92 years) of ARC testing. Conventional cognitive testing was completed at the same time as the clinical evaluation.

Comparing MCs and NCs, there were no significant differences in age, education, self‐reported sex and race, or on smartphone‐based and conventional cognitive testing (all *p's >* 0.05). See Table S1.

### 3.2 Adherence

**FIGURE 1:**
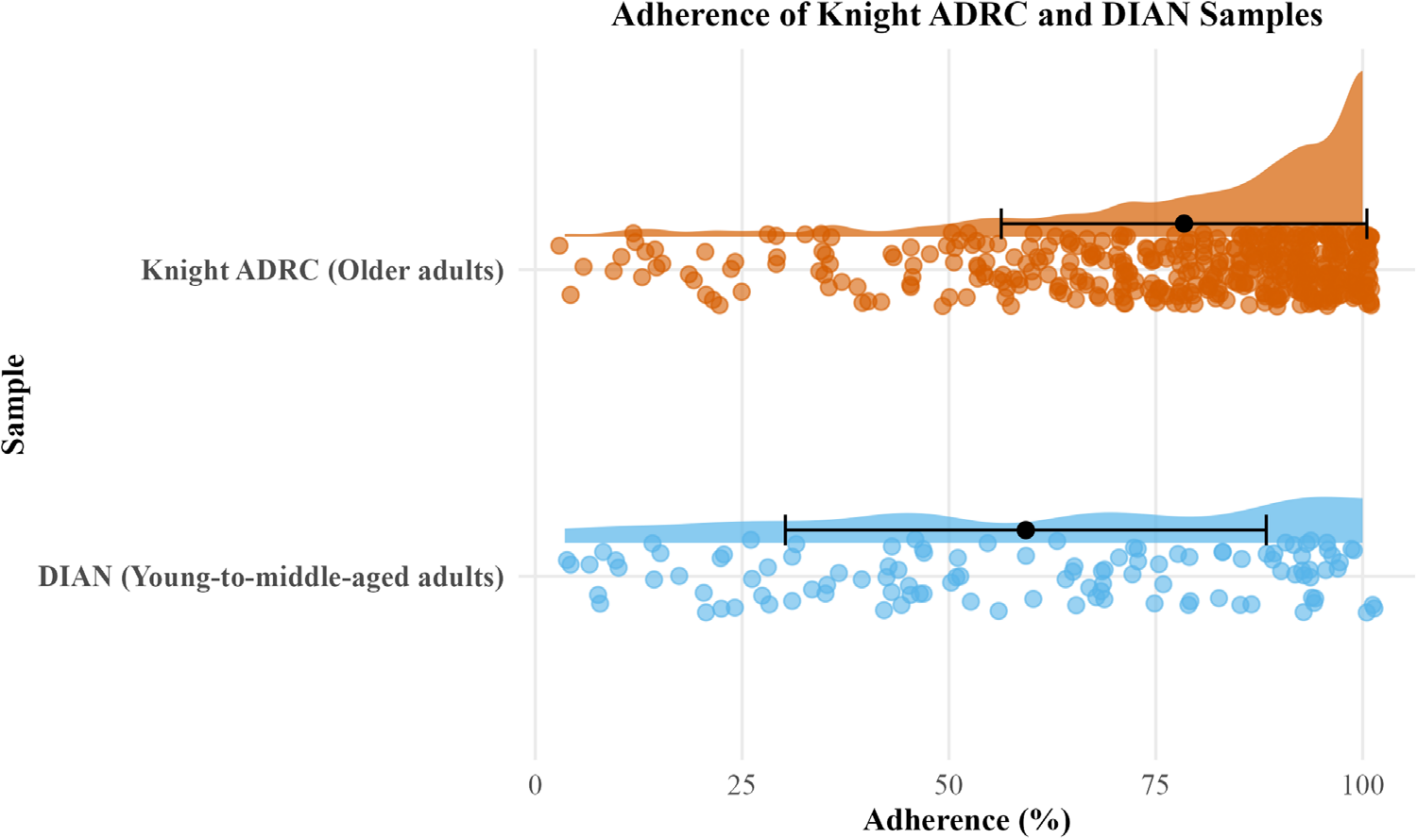
ARC testing adherence of knight ADRC and DIAN samples. The black dot represents the mean adherence for the respective sample. The error bars illustrate the mean +‐ SD. Some of the dots go beyond 100 to prevent overlap of data points. DIAN (*N* = 113), Knight ADRC (*N* = 444). ADRC, Alzheimer Disease Research Center; ARC, Ambulatory Research in Cognition; DIAN, Dominantly Inherited Alzheimer Network observational study.

The Knight ADRC sample completed an average of 78.4 ± 22.1% ARC assessments across the week (Figure 1). This adherence rate is equivalent to completing approximately 22 of 28 sessions. Within the ADRC sample, there was a significant main effect of age on adherence after adjusting for education and sex (β = ‐0.40, 95% confidence interval [CI] [‐0.74, ‐0.06], *SE* = 0.17, *t* = ‐2.29, *p* < 0.05. There was a significant main effect of sex on adherence after adjusting for age and education, such that males had on average 5.59% lower adherence than female participants (β = ‐5.59, 95% CI [‐9.79, ‐1.39], *SE* = 2.14, *t* = ‐2.62, *p* < 0.01. There was no main effect of education on adherence within this sample.

The DIAN sample completed an average of 59.3 ± 29.1% ARC assessments across the week (Figure 1), which is approximately 16 of 28 sessions. Within the DIAN sample, there were no age, sex, or education main effects on adherence. In addition, adherence did not differ between mutation carriers (57%) and non‐carriers (61%), *p *= 0.51.

Central to our main question, when comparing across samples, the Knight ADRC sample had higher adherence than the DIAN sample, after adjusting for education and sex (β = 19.35, 95% CI [14.27, 24.43], *SE* = 2.59, *t* = 7.48, *p* < 0.001). There was a significant main effect of sex on adherence across samples after adjusting for sample and education, such that males had on average 6.14% lower adherence than female participants (β = ‐6.14, 95% CI [‐10.13, ‐2.15], *SE* = 2.03, *t* = ‐3.02, *p *< 0.01). There was no education effect on adherence across samples after adjusting for sample and sex.

An exploratory analysis of time of day and adherence suggested modest time‐of‐day differences between samples. Compared with the DIAN sample, the Knight ADRC sample had a lower probability of completing sessions in the morning (median difference = −0.03, 95% credible interval [−0.05, −0.02], posterior probability of a higher Knight ADRC probability ≈ 0%) and a higher probability in the evening (0.05 [0.02, 0.09], ≈ 99%). Differences were less certain in the afternoon (0.02 [−0.02, 0.06], ≈ 82%) and at night (−0.04 [−0.09, 0.01], ≈ 5%), as both credible intervals included zero.

### 3.3 Reliability

As expected, between‐person reliability of task performance increased with the number of sessions completed across both Knight ADRC and DIAN samples. We pre‐selected 0.90 as the threshold for excellent between‐person reliability. In the Knight ADRC sample, excellent between‐person reliability estimates were attained by five sessions for Symbols, 22 for Grids, and 27 for Prices (See Table S2 for between‐person reliability estimates for up to 28 sessions). Across all ARC tasks, participants attained excellent reliability estimates by 27 sessions. At the average level of adherence of 22 sessions, between‐person reliability estimates of the three tasks were in the good‐to‐excellent range (0.87‐0.98).

In the DIAN sample, excellent between‐person reliability estimates were attained by four sessions for Symbols, 20 for Grids, and 10 for Prices (See Table S2 for between‐person reliability estimates up to 28 sessions). Across all ARC tasks, participants attained excellent reliability estimates by 20 sessions. At the average level of adherence of 16 sessions, between‐person reliability estimates of the three tasks were in the good‐to‐excellent range (0.87–0.97).

**FIGURE 2:**
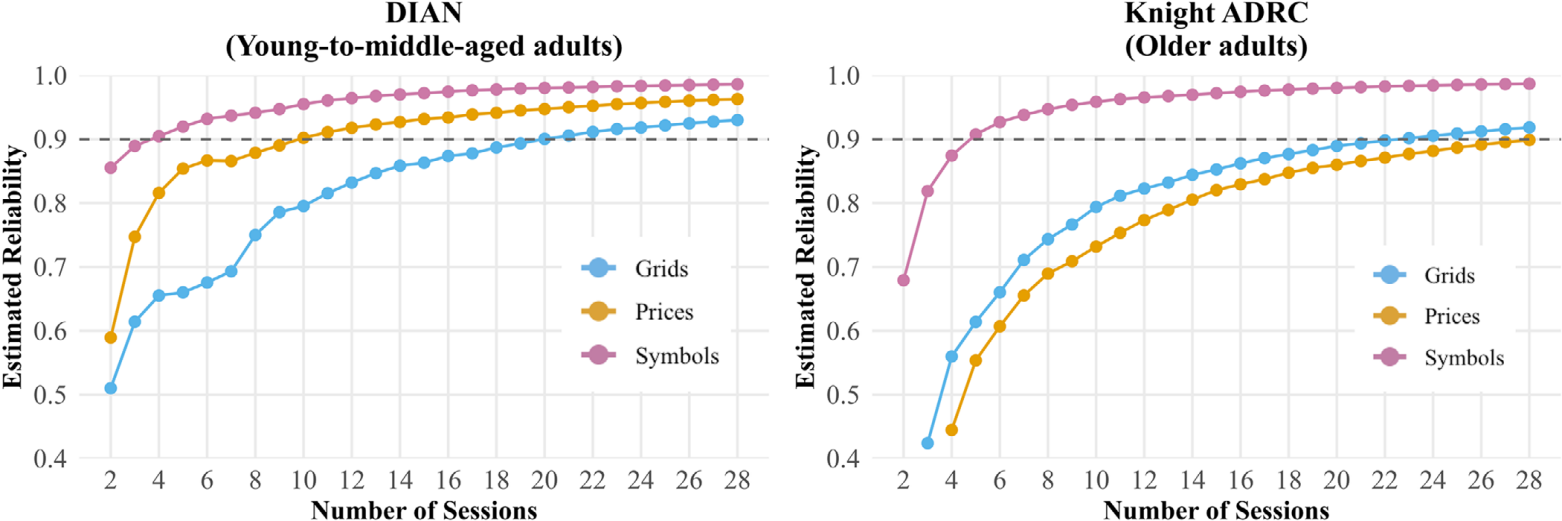
Between‐person reliability estimates across ARC tasks. Between‐person reliability was determined in each sample by estimating the coefficient for each repetition of each of the three ARC tasks. The dashed line represents our predefined goal for maximal between‐person reliability, which was set at 0.90. DIAN (*N* = 113), Knight ADRC (*N* = 444). ADRC, Alzheimer Disease Research Center; ARC, Ambulatory Research in Cognition.

Supporting our hypothesis, comparing the reliability estimates between the samples (Figure 2), the Knight ADRC participants started with considerably lower levels of reliability on the first day of ARC assessments and consistently showed lower reliability across the week. The DIAN sample not only started with higher reliability estimates across the three ARC tasks, these estimates also asymptote more quickly. The DIAN sample achieved excellent between‐person reliability estimates across the three ARC tasks by 20 sessions, while the Knight ADRC sample required more sessions to reach a similar threshold. In the Knight ADRC sample, the Prices subtest had lower reliability throughout the 7‐day assessment cycle, with a maximum estimate of reliability of 0.90 after completing 28 sessions. In the DIAN sample, the Grids subtest had the lowest reliability throughout the week, with a maximum estimate of 0.93 after completing 28 sessions. Across both studies, the Symbols test had exceptional between‐person reliability.

### 3.4 Practice Effects

**FIGURE 3:**
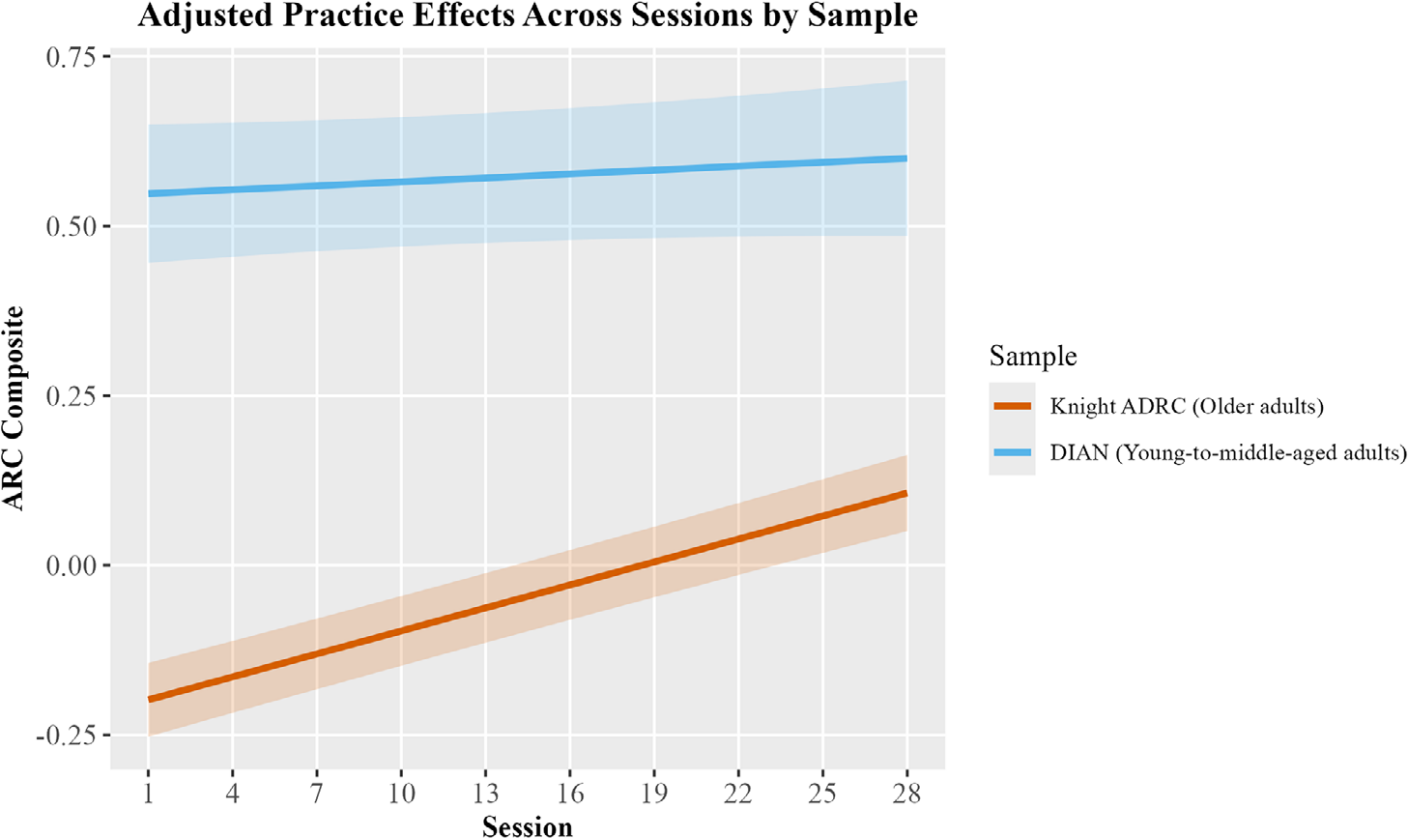
Practice effects for the ARC composite across sessions by sample. Slopes are adjusted for education, sex, total number of testing sessions completed, and the difference in date between the ARC testing and clinical evaluation. DIAN (*N* = 113), Knight ADRC (*N* = 440). ADRC, Alzheimer Disease Research Center; ARC, Ambulatory Research in Cognition; DIAN, Dominantly Inherited Alzheimer Network observational study.

There was a significant interaction between sample and session, indicating that the Knight ADRC participants showed greater improvements on the ARC Composite across the week of testing compared to the DIAN sample, even after accounting for education, sex, number of completed sessions, and the date difference between the ARC testing and clinical evaluation dates (β = ‐0.009, SE = 0.002, *t* = ‐4.60, *p* < 0.001; Figure 3; See Table 3 for the full linear mixed effect model output; See Figure S3 for individual and adjusted practice effects). At session 1, the effect size (the difference between the group estimated marginal means) was ‐0.75, indicating that the ARC composite was 0.75 SD units lower in the Knight ADRC than the DIAN sample, 95% CI [‐0.85, ‐0.64], *p* < 0.0001. At session 28, the effect size was ‐0.49, indicating that the ARC composite was 0.49 SD units lower in the Knight ADRC than the DIAN sample, 95% CI [‐0.62, ‐0.37], *p* < 0.0001. The change in effect size estimates indicates that the Knight ADRC sample improved by approximately 0.25 SD units more than DIAN participants across the week of testing.

**TABLE 3:** Linear mixed effects model predicting the ARC composite across sessions.

Parameter	Estimate	*SE* ^a^	*t*	*p*‐value
Intercept	−1.09	0.14	−7.61	<0.001
Session	0.01	0.0007	14.69	<0.001
DIAN Sample	0.76	0.06	13.61	<0.001
Education	0.04	0.007	5.25	<0.001
Male	−0.08	0.04	−2.02	0.04
Total sessions	0.01	0.003	3.62	<0.001
ARC testing and clinical evaluation date difference	−0.006	0.06	−0.09	0.93
Session*Sample	−0.0094	0.002	−4.60	<0.001

Abbreviations: ARC, Ambulatory Research in Cognition; DIAN, Dominantly Inherited Alzheimer Network observational study; SE, standard error.

The Knight ADRC sample is the reference group in this model. DIAN (*N* = 113), Knight ADRC (*N* = 440).

^a^ SE = standard error.

### 3.5 Correlations with conventional tests

**FIGURE 4:**
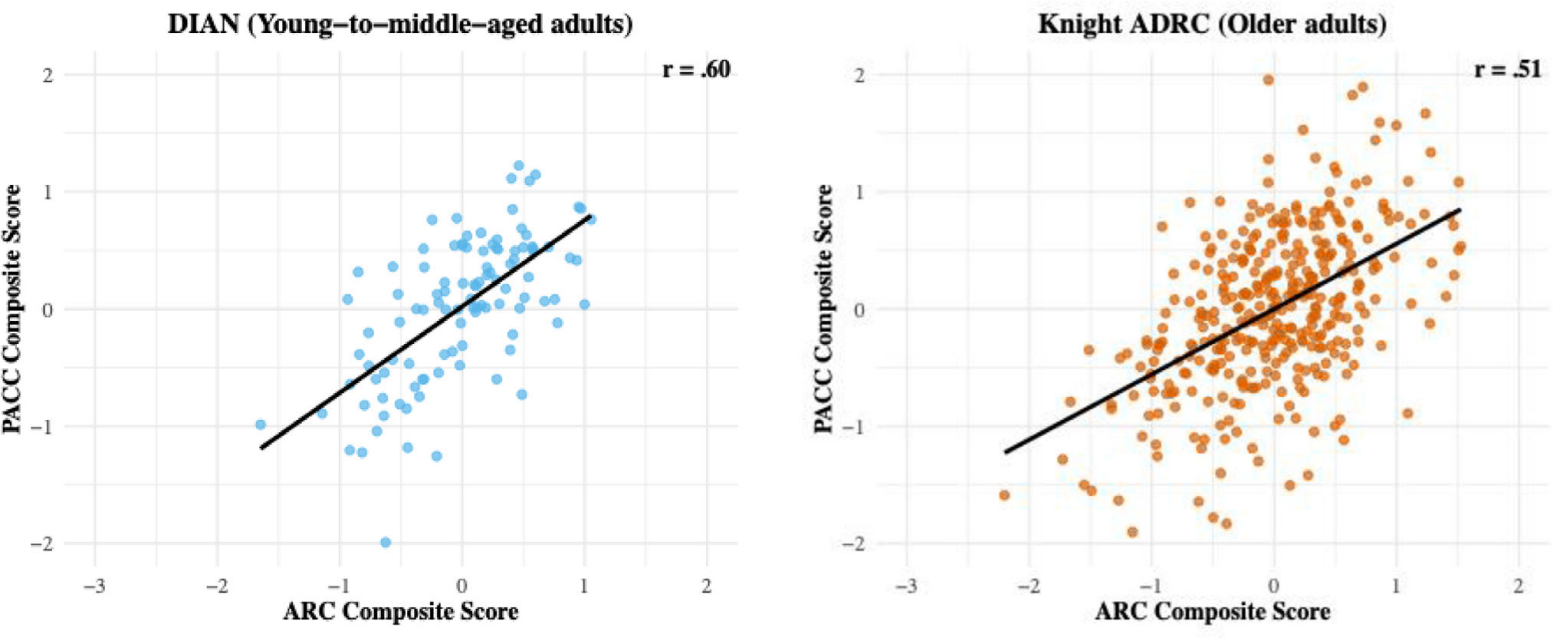
Partial correlation between the ARC and in‐clinic composite scores. A partial correlation is shown for each sample, controlling for education, sex, and the difference in date between the ARC and in‐clinic testing. DIAN (*N* = 107), Knight ADRC (*N* = 366). ADRC, Alzheimer Disease Research Center; ARC, Ambulatory Research in Cognition; DIAN, Dominantly Inherited Alzheimer Network observational study.

In the Knight ADRC sample, there was a strong relationship between the ARC Composite and the Knight‐PACC (*r*(361) = 0.51, 95% CI [0.43, 0.58], *p* < 0.001), controlling for sex, education, and the date difference between the ARC and conventional cognitive testing. Similarly, the DIAN sample showed a strong relationship between the ARC Composite and DIAN‐PACC (*r*(102) = 0.60, 95% CI [0.47, 0.71], *p* < 0.001). While the DIAN sample showed a numerically higher partial correlation, it was not significantly stronger than the Knight ADRC sample (z = ‐1.18, *p* = 0.24; Figure 4).

## 4 DISCUSSION

The objective of this study was to evaluate differences in feasibility and validity of a high‐frequency remote cognitive assessment protocol between two samples: young to middle‐aged adults and older adults at elevated risk for developing AD. The results suggest that remote smartphone‐based cognitive assessments are feasible and valid for both generations of participants. Additionally, this approach may provide increasingly greater utility as older adults become more familiar with smartphone technology. These results have important implications for future studies and especially for the next era of AD prevention trials emphasizing decentralized study designs.

We hypothesized that DIAN participants would have significantly lower levels of adherence compared to the older Knight ADRC participants. This hypothesis was based on prior findings of strong adherence with high‐frequency remote cognitive assessments among older adults^11^ and considering differences in life‐stage demands given the nearly 40‐year age difference between the samples. In line with this hypothesis, the Knight ADRC sample had significantly higher adherence than the DIAN sample (78.4% vs. 59.3%). Our results align with a review of remote smartphone‐based digital health studies that found that older adults tended to have higher engagement and adherence when compared to younger participants.^35^

There are several possible additional explanations for the higher adherence among the Knight ADRC sample. Based on concerns regarding technology familiarity in older adults, the ARC study in the Knight ADRC was designed to include frequent support from study coordinators to inquire about technological issues or when adherence rates fell below 60%.^8^ In DIAN, this type of frequent contact and technical support was more difficult to manage and coordinate, as DIAN is a multi‐site, international study where DIAN coordinators are asked to manage all aspects of data collection. This emphasizes the importance of maintaining contact with all participants to ensure high adherence in studies that rely on high‐frequency remote cognitive assessments.

Another possible interpretation is that engaging with the repeated testing on the ARC platform may serve as a form of disclosure of AD risk. Prior work suggests that individuals are less open to learning about research results when they perceive greater risk of developing the disease.^36^, ^37^ Notably, many DIAN participants remain unaware of their genetic status, often by choice.^38^ Thus, future studies should conduct participant interviews to inquire whether self‐evaluation of repeated engagement with ARC tasks possibly contributes to lower adherence on high‐frequency cognitive testing among those with a family history of DIAD regardless of awareness of genetic status.

The exploratory analysis provides preliminary support for potential differences in life‐stage demands between young‐to‐middle‐aged and older adults. The DIAN participants were more likely to complete more sessions in the morning and fewer sessions in the evening than the Knight ADRC sample. Adherence to high‐frequency cognitive assessment among young‐to‐middle‐aged adults may be improved if this age group is allowed to complete a greater number of sessions in the morning. This could look like increasing the current 7‐day testing to a greater number of days.

The hypothesis that the DIAN participants would demonstrate higher levels of between‐person reliability estimates than the Knight ADRC participants was supported. The results indicated that the Knight ADRC and DIAN samples achieved excellent reliability across all ARC tests by the end of the week of testing. The Knight ADRC participants achieved a specified goal of ≥ 0.90 reliabilities by 27 sessions, while the DIAN participants did so by just 20 sessions. These results for older adults are similar to another high‐frequency cognitive assessment study^32^ that found that several repeated sessions were required to reach similar reliability thresholds in older adults. Additionally, a recent paper^39^ found between‐person reliability in middle‐aged participants to exceed 0.80 with fewer than 10 repeated sessions on attentional control and processing speed tasks, which aligns with our findings in DIAN participants.

Ideally, high‐frequency cognitive assessments are designed so that the maximum level of reliability can be achieved with the fewest number of repetitions. Although many studies in the high‐frequency assessment literature refer to 80% as an acceptable rate of adherence, the rationale for this threshold is debated.^40^, ^41^ Some argue that 80% adherence is acceptable for self‐report data (not objective cognitive data) and would allow for more generalizable conclusions about participants’ daily lives.^42^ It is our position that the minimum rate of adherence for high‐frequency cognitive assessments should be informed by between‐person reliability. In our study, despite lower adherence, the DIAN sample achieved excellent reliabilities with fewer completed sessions than the Knight ADRC sample. In contrast, the Knight ADRC sample required more sessions to achieve the same 0.90 threshold, but this was feasible given their higher adherence. Thus, if the goal of a study is to examine aggregated weekly outcomes, then fewer completed sessions may be sufficient in younger populations, like the DIAN sample. The rationale for attempting 28 sessions in 1 week was driven by the few existing studies using high‐frequency paradigms to assess cognition (e.g., Sliwinski et al., 2018) when ARC was developed, but also by our interest in other outcomes that can be acquired from high‐frequency assessments, including learning rates^43^, ^44^ and variability at different timescales.^45^, ^46^ Such analytic goals may require a greater number of sessions completed. Thus, the optimal assessment frequency should reflect the goals of the study.

The Knight ADRC sample demonstrated significantly greater practice effects than the DIAN sample. However, this finding is not consistent with a meta‐analysis suggesting practice effects in cognitive assessment are reduced in older adults. Calamia et al. (2012) theorize that practice effects should become smaller with increasing age because older adults fail to encode test stimuli or useful strategies for improving in subsequent assessments.^47^ Indeed, we have observed attenuated practice effects associated with dementia onset in healthy older adults on paper‐and‐pencil cognitive measures in the Knight ADRC.^48^ However, practice effects in a cognitively normal sample may become greater with increasing age when assessing cognition with smartphone‐based formats. The larger practice effects among the Knight ADRC participants may demonstrate increasing familiarity with the smartphone interface across sessions rather than true cognitive change. Additionally, ARC tasks emphasize speed of performance and have brief, time‐restricted learning phases. The Knight ADRC participants likely had a lower baseline of technology familiarity and were approximately 45% slower, on average, on the Symbols processing speed task. Together, these likely resulted in a steeper learning curve for older adults. This is a possible explanation for why the older Knight ADRC sample required more repetitions to achieve similar levels of between‐person reliability as the younger DIAN sample. In other words, older adults may simply require more repetitions to acclimate to a smartphone‐based format. In contrast, the DIAN sample demonstrated almost no detectable practice effects on the ARC measures likely due to faster processing speed and greater digital fluency, which may also explain why the younger DIAN participants achieved excellent between‐person reliability with fewer sessions.

Contrary to our hypothesis, the correlations between the ARC tests and conventional, in‐clinic cognitive tests were not significantly stronger in the DIAN sample compared to the Knight ADRC sample. However, both the Knight ADRC and DIAN samples showed a strong relationship between the ARC and in‐clinic tests. This strong association for the DIAN participants was evident despite this sample completing fewer assessments on average. Our results indicate that high‐frequency cognitive testing is valid among both young‐to‐middle‐aged adults and older adults.

There are several limitations to consider. The primary interpretation of the differences between the two samples centers on generational differences in technology familiarity, but we lack a formal assessment of this in all participants. Second, sample sizes differed greatly; DIAD is extremely rare, and enrolling participants in an international study was challenging. Third, the international DIAN sample inherently has greater cultural and educational heterogeneity compared to the more homogeneous Knight ADRC sample. Future studies exploring age differences in high‐frequency smartphone‐based cognitive testing should consider comparing homogeneous samples in terms of cultural, socioeconomic, and educational backgrounds if a large enough sample size is available. Controlling for these factors may strengthen the concept of age‐related technology familiarity differences occurring with this testing modality. Finally, we have limited information on reasons for lower adherence in DIAN, so interpretations about life‐stage demands and risk disclosure avoidance could not be verified.

## 5 Conclusions

This study demonstrates that high‐frequency smartphone‐based cognitive assessments are feasible and valid across adulthood among individuals at risk for AD. Older Knight ADRC participants exhibited higher adherence, likely due to structured support. The younger DIAN participants engaged less overall, yet achieved greater reliability with fewer sessions, showed smaller practice effects, and demonstrated strong correlations between smartphone‐based and in‐clinic cognitive measures, reflecting stronger digital fluency. These findings suggest that younger adults may require fewer remote sessions without compromising reliability or validity, while older adults benefit from more frequent testing to offset practice effects and increase reliability. Importantly, older participants’ strong adherence supports the feasibility of this design for their age group. Generational differences appear to influence both participation and data quality; however, as the digital divide continues to narrow, remote cognitive testing is poised to become increasingly effective, enabling decentralized designs in future AD prevention trials.

## CONFLICT OF INTEREST STATEMENT

Andrew Aschenbrenner received consulting fees from CognitionMetrics LLC. Takeshi Ikeuchi received consulting fees from FujiRebio Inc, Sysmexs, Eisai, Eli Lilly, Roche Diagnostics. He received payment or honoraria from Alector, Alnylum, Eisai, Eli Lilly, PDR Pharma, Roche Diagnostics, Kowa, and Chugai. Eric McDade received consulting fees from Astra Zeneca, Roche, Sanofi, Merck, Quidel‐Ortho Diagnostics, Voyager. Payment or honoraria for lectures from Projects in Knowledge (Kaplan)‐ CME, Neurology Live‐ CME. Travel support from the Alzheimer Association and Fondation Alzheimer. He is paid royalties for the following patents: T‐018562. He holds a leadership or fiduciary role in Alazamend. Receipt of equipment, materials, drugs, medical writing, gifts or other services include Avid Radiopharmaceuticals, Cerveau, LMI. Gregory S Day reports payments or honoraria from PeerView Media, Continuing Education, Inc., DynaMed, Ionis Pharma, MJH Life Sciences. Receipt of equipment, materials, drugs, medical writing, gifts or other services include Amgen Therapeutics and payment was made towards material support of clinical trial (NCT04372615) and AVID radiopharmaceuticals and payment was made towards material support of radiotracer for research. Johannes Levin reports speaker fees from Bayer Vital, Biogen, EISAI, TEVA, Zambon and Roche, consulting fees from Axon Neuroscience, EISAI and Biogen, author fees from Thieme medical publishers and W. Kohlhammer GmbH medical publishers, and is the inventor in a patent [EP 23 156 122.6] filed by LMU Munich. In addition, he reports compensation from MODAG GmbH, is beneficiary of the phantom share program of MODAG GmbH and is inventor in a patent [EP 22 159 408.8] filed by MODAG GmbH, all activities outside the submitted work. Tammie LS Benzinger received consulting fees from Biogen, Eli Lilly and Company, Eisai, Bristol Myers Squibb, Johnson & Johnson, and Merck & Co., Roche. She received payments or honoraria from Medscape, PeerView, Neurology Today, Med Learning Group, and Applied Radiology. Travel support from Cedars‐Sinai Medical Center, Hong Kong Neurological Association, Alzheimer's Association, American College of Radiology, Radiological Society of North America, Stanford University, Johnson & Johnson. Patents include US Patent 16/097, US Patent 12,016,701. Participation on a Data Safety Monitoring Board or Advisory Board include: Siemens Advisory Board. Neither John C. Morris nor his family owns stock or has equity interest (outside of mutual funds or other externally directed accounts) in any pharmaceutical or biotechnology company. Chengjie Xiong reports consulting fees from Diadem. Randall J. Bateman is a co‐inventor on the following US patent applications: “Methods to detect novel tau species in CSF and use thereof to track tau neuropathology in Alzheimer's disease and other tauopathies” and “CSF phosphorylated tau and amyloid beta profiles as biomarkers of Tauopathies”. He is also a co‐inventor on a non‐provisional patent application: “Methods of diagnosing and treating based on site‐specific tau phosphorylation”. He receives research funding from Avid Radiopharmaceuticals, Janssen, Roche/Genentech, Eli Lilly, Eisai, Biogen, AbbVie, Bristol Myers Squibb and Novartis. Jason Hassenstab reports consulting fees from Prothena, AlzPath, Quanterix, and Abbvie. All other authors report no conflicts of interest. Author disclosures are available in the Supporting Information.

## CONSENT STATEMENT

All Knight ADRC participants provided informed consent, and all procedures were approved by the Human Research Protections Office at Washington University in St. Louis. Before the completion of any DIAN procedures, all DIAN participants provided informed consent in accordance with the local institutional review boards.

## Supporting information



Supporting Information

Supporting Information

Supporting Information

Supporting Information
