# Supplementary material for: Technology‐natives and technology‐adopters: Age differences in remote cognitive assessment in Alzheimer disease
Source: Alzheimers Dement. 2026 Sep 24;22(9):e71872. doi: 10.1002/alz.71872 (PMC13602171; doi:10.1002/alz.71872)
Supplement: Supplementary file 2 [file ALZ-22-e71872-s001.docx]

Supplemental Table 2:

Knight ADRC, DIAN Samples: Between-persons Reliability Estimates for ARC Tasks

| **Knight ADRC Sample** | | | |
| --- | --- | --- | --- |
| **Sessions** | **Symbols** | **Grids** | **Prices** |
| 2 | 0.679 | 0.247 | 0.160 |
| 3 | 0.819 | 0.424 | 0.328 |
| 4 | 0.874 | 0.560 | 0.445 |
| 5 | 0.907 | 0.614 | 0.554 |
| 6 | 0.927 | 0.660 | 0.607 |
| 7 | 0.938 | 0.711 | 0.655 |
| 8 | 0.947 | 0.744 | 0.690 |
| 9 | 0.954 | 0.766 | 0.709 |
| 10 | 0.959 | 0.794 | 0.732 |
| 11 | 0.963 | 0.811 | 0.753 |
| 12 | 0.965 | 0.823 | 0.773 |
| 13 | 0.968 | 0.832 | 0.789 |
| 14 | 0.970 | 0.844 | 0.805 |
| 15 | 0.972 | 0.853 | 0.820 |
| 16 | 0.974 | 0.862 | 0.829 |
| 17 | 0.976 | 0.870 | 0.837 |
| 18 | 0.978 | 0.877 | 0.848 |
| 19 | 0.979 | 0.883 | 0.855 |
| 20 | 0.980 | 0.889 | 0.860 |
| 21 | 0.982 | 0.894 | 0.866 |
| 22 | 0.983 | 0.898 | 0.871 |
| 23 | 0.983 | 0.902 | 0.877 |
| 24 | 0.984 | 0.905 | 0.882 |
| 25 | 0.985 | 0.909 | 0.887 |
| 26 | 0.986 | 0.912 | 0.892 |
| 27 | 0.986 | 0.916 | 0.895 |
| 28 | 0.987 | 0.918 | 0.899 |
| **DIAN Sample** | | | |
| **Sessions** | **Symbols** | **Grids** | **Prices** |
| 2 | 0.855 | 0.510 | 0.589 |
| 3 | 0.889 | 0.614 | 0.747 |
| 4 | 0.905 | 0.655 | 0.816 |
| 5 | 0.920 | 0.660 | 0.854 |
| 6 | 0.932 | 0.676 | 0.867 |
| 7 | 0.937 | 0.693 | 0.866 |
| 8 | 0.941 | 0.750 | 0.879 |
| 9 | 0.947 | 0.786 | 0.890 |
| 10 | 0.955 | 0.795 | 0.902 |
| 11 | 0.961 | 0.815 | 0.911 |
| 12 | 0.964 | 0.832 | 0.918 |
| 13 | 0.968 | 0.847 | 0.923 |
| 14 | 0.970 | 0.859 | 0.927 |
| 15 | 0.972 | 0.863 | 0.932 |
| 16 | 0.974 | 0.874 | 0.934 |
| 17 | 0.977 | 0.878 | 0.939 |
| 18 | 0.978 | 0.887 | 0.941 |
| 19 | 0.979 | 0.893 | 0.945 |
| 20 | 0.980 | 0.900 | 0.947 |
| 21 | 0.981 | 0.906 | 0.950 |
| 22 | 0.982 | 0.911 | 0.952 |
| 23 | 0.983 | 0.916 | 0.955 |
| 24 | 0.983 | 0.918 | 0.957 |
| 25 | 0.984 | 0.922 | 0.959 |
| 26 | 0.985 | 0.925 | 0.960 |
| 27 | 0.985 | 0.928 | 0.962 |
| 28 | 0.986 | 0.930 | 0.963 |
