# Supplementary material for: Technology‐natives and technology‐adopters: Age differences in remote cognitive assessment in Alzheimer disease
Source: Alzheimers Dement. 2026 Sep 24;22(9):e71872. doi: 10.1002/alz.71872 (PMC13602171; doi:10.1002/alz.71872)
Supplement: Supplementary file 3 [file ALZ-22-e71872-s002.docx]

*Supplemental Figure 1. Knight ADRC Sample- Demographic, ARC, and In-Clinic Pearson Correlations*

*Note.* Pairwise correlations have different sample sizes based on availability of data. Age, Education, Prices, Adherence (N = 444); ARC Composite, Grids, Symbols (N = 443); Category Fluency Animals (N = 442); SRT Free Recall, Number Symbol (N = 381); Knight-PACC Composite (N = 366); Trail Making Test Part B (N = 135).

*Supplemental Figure 2. DIAN Sample- Demographic, ARC, and In-Clinic Pearson Correlations*

*Note.* Pairwise correlations have different sample sizes based on availability of data. Age, Education, Estimated Years to Symptom Onset, ARC Composite, Grids, Prices, Symbols, Adherence (N = 113); DIAN-PACC, Category Fluency Animals, Digit Symbol (N = 107); Trail Making Test Part B (N = 105); Logical Memory II Delayed Recall (N = 98).

*Supplemental Figure 3: Individual and Adjusted Practice Effects for the ARC Composite Across Sessions by Sample*

Spaghetti plot illustrating individual participant trajectories of the ARC Composite across the week. Slopes are adjusted for education, sex, total number of testing sessions completed, and the difference in date between the ARC testing and clinical evaluation. DIAN (N = 113), Knight ADRC (N = 440).
