# Supplementary material for: Technology‐natives and technology‐adopters: Age differences in remote cognitive assessment in Alzheimer disease
Source: Alzheimers Dement. 2026 Sep 24;22(9):e71872. doi: 10.1002/alz.71872 (PMC13602171; doi:10.1002/alz.71872)
Supplement: Supplementary file 4 [file ALZ-22-e71872-s003.docx]

Supplemental Table 1: DIAN Sample Demographic Data by Mutation Status

|  | Not Mutation Carrier  N = 57*^1^* | Mutation Carrier  N = 56*^1^* | p-value*^2^* |
| --- | --- | --- | --- |
| Age | 36.80 (10.48) | 34.70 (10.19) | 0.28 |
| Sex (% Female)*^3^* | 36 (63.2%) | 32 (57.1%) | 0.64 |
| Race*^3^* |  |  | 0.60 |
| Black | 11 (19.3%) | 6 (10.7%) |  |
| White | 41 (71.9%) | 46 (82.1%) |  |
| Other | 5 (8.8%) | 4 (7.1%) |  |
| Education | 14.68 (2.89) | 14.50 (2.93) | 0.74 |
| Estimated Year to Onset (EYO) | -15.01 (13.05) | -15.08 (8.79) | 0.97 |
| Grids | 0.52 (0.27) | 0.50 (0.24) | 0.76 |
| Prices | 0.26 (0.14) | 0.25 (0.13) | 0.59 |
| Symbols | 2.16 (0.67) | 2.38 (0.76) | 0.10 |
| Standardized ARC Composite | 0.17 (0.57) | 0.16 (0.63) | 0.90 |
| Adherence | 61.1% (28.4%) | 57.46% (29.8%) | 0.51 |
| Logical Memory II Delayed recall | N = 50  13.16 (4.77) | N = 48  13.60 (4.05) | 0.62 |
| Category Fluency Animals | N = 55  21.00 (6.27) | N = 52  22.13 (6.29) | 0.34 |
| Digit Symbol | N = 55  57.89 (18.36) | N = 52  61.92 (16.14) | 0.23 |
| Trail Making Test Part B | N = 54  65.57 (44.29) | N = 51  61.29 (26.68) | 0.55 |
| Standardized In-clinic Composite | N = 55  0.04 (0.75) | N = 52  0.19 (0.58) | 0.24 |

*^1^Mean (SD); n (%)*

*^2^Welch Two Sample t-test; Pearson’s Chi-squared test*

*^3^Sex and race were self-reported*
